# Comparison of myocardial infarct size measurements between noncontact mapping and cardiac contrast enhanced MRI

**DOI:** 10.1186/1532-429X-11-S1-P90

**Published:** 2009-01-28

**Authors:** Henning Steen, Frederik Voss, Rüdiger Becker, Hugo A Katus, Evangelos Giannitsis

**Affiliations:** University of Heidelberg, Heidelberg, Germany

**Keywords:** Infarction Size, Cardiac Magnetic Resonance Imaging, Myocardial Infarct Size, Magnetic Resonance Imaging Measurement, Significant Linear Correlation

## Introduction

Once chamber geometry is determined, the EnSite 3000 noncontact mapping system can create a voltage map during a single cardiac cycle. The EnSite uses an inverse solution to the Laplace equation to process the amplified far-field signals from the noncontact catheter. This process creates a three-dimensional endocardial potential map from a single cardiac cycle. Dynamic substrate mapping (DSM) is an algorithm designed to identify conduction boundaries, such as myocardial scars based on voltage distribution within the corresponding chamber.

## Purpose

The purpose of this study was to investigate the correlation between DSM- and magnetic resonance imaging (MRI) – determined scar areas and to identify a suitable DSM voltage threshold.

## Methods

A total of eight dogs were studied. Four healthy foxhounds underwent ligation of the left anterior descending coronary artery. Evidence of myocardial infarction, including ECG changes and elevated cardiac troponin T levels, was noted in all animals. Cardiac MRI scan was performed 29 ± 2 days after ligation of the left anterior descending coronary artery. Subsequently, noncontact mapping of the left ventricle was obtained in each dog, and myocardial infarction size was determined using DSM at different filter settings. As a control group, another four foxhounds underwent sham thoracotomy/pericardiotomy.

## Results

A significant linear correlation of infarction size using DSM compared with MRI measurements was found at the filter setting "peak negative 34%" (P = 0.001, r = 0.99). Mean relative infarction size was 15.9% ± 4.5% with DSM and 16.0% ± 4.2% with MRI. Compared with the sham group, a significant reduction in left ventricular ejection fraction was found after ligation of the left anterior descending coronary artery (51.0% ± 3.8% vs 69.2% ± 5.9%, P = 0.002). Pathoanatomic studies were performed to confirm the measured infarct dimensions. No scars were detectable in shamoperated dogs using DSM or MRI.

## Conclusion

Noncontact mapping allows identification of scar tissue within the left ventricle. An excellent correlation was observed between DSM-scar surface and MRI-determined scar size. Identifying and marking these areas can be useful when planning an ablation strategy in the clinical setting of ischemic heart disease.

